# Suppressing the Substance P-NK1R Signalling Protects Mice against Sepsis-Associated Acute Inflammatory Injury and Ferroptosis in the Liver and Lungs

**DOI:** 10.3390/antiox13030300

**Published:** 2024-02-29

**Authors:** Zhixing Zhu, Stephen Chambers, Madhav Bhatia

**Affiliations:** 1 Department of Pathology and Biomedical Science, University of Otago, Christchurch 8140, New Zealand; zhixing.zhu@postgrad.otago.ac.nz (Z.Z.); steve.chambers@otago.ac.nz (S.C.); 2 Department of Internal Medicine, Teaching and Research Section, The Second Clinical Medical School of Fujian Medical University, Quanzhou 362002, China

**Keywords:** substance P, neurokinin-1 receptor, ferroptosis, liver injury, lung injury, sepsis

## Abstract

Substance P (SP), encoded by the *TAC1/Tac1* gene, acts as a significant mediator in dysregulated systemic inflammatory response and associated organ injury in sepsis by activating the neurokinin-1 receptor (NK1R). This study investigated the impact of SP-NK1R signaling on ferroptosis in the liver and lungs of mice with sepsis. Sepsis was induced by caecal ligation puncture (CLP) surgery in mice. The SP-NK1R signaling was suppressed by *Tac1* gene deletion, NK1R blockade, and a combination of these two approaches. The physiological conditions of mice were recorded. The profile of the SP-NK1R cascade, inflammatory response, ferroptosis, and tissue histology were investigated in the liver and lungs. Several manifestations of sepsis occurred in *Tac1*^+/+^ mice during the development of sepsis. Notably, hypothermia became significant four hours after the induction of sepsis. In the liver and lungs of mice subjected to CLP surgery, the concentrations of SP and NK1R were upregulated. Additionally, the concentrations of pro-inflammatory mediators, including cytokines (IL-1β, IL-6, and TNF-α) and chemokines (MCP-1 and MIP-2), were increased. Moreover, ferroptosis was elevated, as evidenced by increased concentrations of iron and MDA and reduced concentrations of GSH, Nrf2, and Gpx4. Suppressing the SP-NK1R cascade significantly mitigated CLP-surgery-induced alterations in mice. Importantly, these three approaches used to suppress SP-NK1R signaling showed similar effects on protecting mice against sepsis. In conclusion, increased SP-mediated acute inflammatory response and injury in the liver and lungs in mice with CLP-surgery-induced sepsis was associated with elevated ferroptosis. The detrimental effect of SP on sepsis was predominantly mediated by NK1R. Therefore, the suppression of increased SP-NK1R signaling and ferroptosis may be a promising adjuvant therapeutic candidate for sepsis and associated acute liver and lung injury.

## 1. Introduction

Sepsis, a major international health challenge, refers to a life-threatening pathological condition characterised by organ impairment induced by a dysregulated immune system [1,2]. The physiopathology by which the dysregulations of acute inflammatory response and associated organ injury occur in sepsis is yet to be investigated in depth [3].

Substance P (SP) is an undecapeptide encoded by the *Tac1* gene in rodents [4]. It has been implicated in diverse biological processes and pathological settings, predominantly by activating its functional receptor, named neurokinin 1 receptor (NK1R) [5]. Increased SP-NK1R signalling has been shown to contribute to aberrant systemic inflammatory response, leading to acute inflammatory injury in multiple organs, including the liver, lungs, and kidney in sepsis [5,6,7]. However, the precise mechanism still needs to be fully understood.

Emerging evidence has highlighted the involvement of unorganized programmed cell death in the host acute inflammatory response in sepsis [8]. Ferroptosis is a newly identified programmed cell death triggered by intracellular iron-overload-mediated lipid peroxidation [9,10,11]. Ferroptosis is closely related to damage-associated molecular patterns and the release of pro-inflammatory mediators, which further results in increased host acute inflammatory responses [11,12,13]. It has been recognized as a significant contributor to the pathogenesis of host-dysregulated acute inflammatory response in sepsis and associated organ injury [14,15,16]. The process of ferroptosis is modulated by multiple signalling cascades [9,10,11]. Glutathione peroxidase 4 (Gpx4) is an important inhibitor of ferroptosis that uses glutathione (GSH) as a reducing substrate to inhibit lipid peroxidation and maintain lipid homeostasis [17,18]. The transcription factor nuclear factor erythroid 2-related factor 2 (Nrf2) has also been recognized as a versatile negative regulator of ferroptosis [19,20]. Nrf2 can modulate iron recycling, storage, and usage and is therefore central to iron homeostasis. Nrf2 can also increase the biosynthesis of GSH and enhance the expression and activity of Gpx4 in repressing lipid peroxidation [19,20]. Consequently, the dysregulation of the Nrf2-Gpx4 cascade has been recognized as an important mechanism of ferroptosis in a wide range of disorders [21,22,23]. Under pathological conditions, ferroptosis and host acute inflammatory response can lead to each other, thus driving an auto-amplification loop and further exaggerating ferroptosis and the host acute inflammatory response [12,24,25]. Therefore, the dysregulated activation of pro-inflammatory signalling pathways, such as the NF-κB signalling pathway and MAPK signalling pathway, leading to increased acute inflammatory response, has also been recognised as a significant mechanism of aberrant ferroptosis in various diseases [12,24,25].

The activation of the ERK1/2-NF-κB cascade promotes the pro-inflammatory role of SP in CLP-induced sepsis and organ damage [26]. Moreover, previous studies have shown that SP upregulated the expression of cyclooxygenase-2 (COX-2), a well-recognized driver and marker of ferroptosis [11], in various pathological conditions [27,28,29]. Noteworthily, increased SP-NK1R signalling has been shown to increase the expression of COX-2 in the lungs of mice following local burn injury via the activation of the ERK1/2-NF-κB cascade [27]. Considering the roles of the ERK1/2-NF-κB cascade and COX-2 in ferroptosis and that of the SP-NK1R axis in activating the ERK1/2-NF-κB cascade and promoting the expression of COX-2, increased SP-NK1R signalling may also promote ferroptosis in sepsis.

However, whether the SP-NK1R axis contributes to sepsis and associated organ injury via the promotion of ferroptosis remains to be investigated in depth. Likewise, whether dysregulated ferroptosis is involved in the impacts of the SP-NK1R axis on acute inflammatory response and organ injury in mice with sepsis is not fully understood. Therefore, this study aimed to investigate the potential impact and underlying mechanism of endogenous SP on ferroptosis and associated acute inflammatory injury in the liver and lungs in mice following caecal ligation puncture (CLP)-surgery-induced sepsis. To achieve this aim, the genetic deletion of the *Tac1* gene, the pharmacological blockade of NK1R using L703606, and the combination of these two approaches were employed in this study.

## 2. Materials and Methods

### 2.1. Group Setting

**Figure 1:**
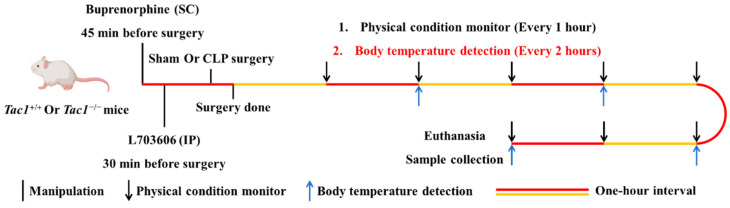
Flowchart of experimental design including time course of manipulations, physical condition records, body temperature monitor, euthanasia, and sample collection in mice.

This in vivo study is regulated by the Animal Welfare Act and was approved by the University of Otago Animal Ethics Committee (AUP-19-104). The protocol used in this in vivo study is presented in Figure 1. Twenty-four *Tac1*^+/+^ (Balb/c, male) and twenty-four *Tac1*^−/−^ (Male) mice aged between 8 and 10 weeks with body weights ranging from 20 to 30 g were randomly divided into three groups of eight mice: the sham-saline group, the CLP-saline group, and the CLP-L703606 group, respectively (n = 8 each). *Tac1*^+/+^ mice were acquired from the Christchurch Animal Research Area. *Tac1*^−/−^ mice were a gift from Prof. AI Basbaum (University of California, San Francisco, CA, USA) and bred as described previously [30,31].

### 2.2. Sepsis Establishment, Manipulations, and Sample Collection in Mice

CLP-surgery-induced sepsis was induced according to a previously reported protocol with minor modifications [32]. L703606, a specific antagonist of NK1R widely used to block the interaction between SP and NK1R, was dissolved in DMSO and then diluted in sterile saline before experiments. Then, 0.2 mL of saline or L703606 (4 mg/kg) was peritoneally injected into all mice 30 min before the sham or CLP surgery. Prior to surgery, mice were anesthetized by inhaling 2% isoflurane. During the operation, inhaled isoflurane was reduced to 1%. Aseptic surgical techniques were applied during surgery. Firstly, the abdominal area was disinfected after the abdominal fur was shaved. Secondly, a small midline incision was made through the skin and peritoneum of the abdomen to expose the caecum. Thirdly, the cecum was ligated at the designated position (1 cm from the tip of the cecum) without occluding the bowel passage with silkam 5.0. Subsequently, the cecum was perforated at the distal end using a 22-gauge needle to make a through-and-through puncture. Afterwards, a small amount of stool was squeezed out through both holes. Thereafter, the bowel contents were repositioned and the abdomen was sutured using permilene 5.0 thread. Mice in the sham operation group underwent the same operation without CLP procedure. All mice received saline at the end of the operation by the subcutaneous route. Buprenorphine (Temgesic, 0.2 mg/kg) was subcutaneously injected into all mice 45 min before and 3 h after surgery for analgesia. Eight hours after the sham or CLP surgery, mice were euthanized by intraperitoneally injecting a lethal dose of sodium pentobarbital (150 mg/kg). Blood was withdrawn from the right ventricle using heparinized syringes via cardiac puncture and then spun for 5 min at 4 °C (1000 g). Then, the plasma was carefully pipetted into chilled microtubes and then kept in a −80 °C freezer. The liver tissues were either fixed in 10% neutral phosphate-buffered formalin or frozen in liquid nitrogen and further transferred to a −80 °C freezer. The cranial lobe of the right lung was used for the wet-to-dry (W/D) ratio measurement. The remaining lung tissues were processed and stored as for the protocol applied to the liver tissues.

### 2.3. Evaluation of Sepsis Severity in Mice

The physical condition of mice after the sham or CLP operation was recorded hourly for eight hours. The body temperature was detected in four randomly selected mice in each group through a rectal probe every two hours for eight hours. The severity of sepsis was evaluated according to a previously reported murine sepsis scoring system with minor modifications [33].

### 2.4. Measurement of Lung W/D Ratio

The cranial lobe of the right lung from every mouse was dissected and weighed immediately (wet weight). Every collected lobe was kept in a small cassette and placed in a 60 °C oven for 24 h. On the second day, these lobes were weighed again (dry weight). The lung W/D ratio was calculated as lung W/D ratio = wet weight/dry weight.

### 2.5. Determination of SP in Tissues

Tissue samples were weighted (~20 mg), thawed, snipped, homogenized in 20 mM phosphate buffer (pH 7.4), and spun (4 °C, 10,000× *g*, 10 min). Supernatants were transferred to fresh chilled microtubes. The concentrations of SP in the supernatants were measured using the competitive ELISA method according to the instruction of the SP ELISA kit (Cayman Chemical, Ann Arbor, MI, USA). The concentration of protein in tissues was determined by the Bradford assay. The concentration of SP was corrected for the total protein content of tissues and expressed as ng per mg protein.

### 2.6. Measurement of Pro-Inflammatory Cytokines and Chemokines in Tissues

The concentrations of cytokines (IL-1β, IL-6, and TNF-α) and chemokines (MCP-1 and MIP-2) in the liver and lungs were measured using the sandwich ELISA method according to the instructions of the DuoSet ELISA kits (R&D System, Minneapolis, MN, USA). The concentrations of these mediators in tissues were corrected for the total protein content of the tissues. The results were presented as pg or ng per mg protein.

### 2.7. Western Blotting

Tissues were homogenized in chilled RIPA buffer supplemented with protease and phosphatase inhibitors on ice. Homogenates were then centrifuged for 10 min (4 °C, 15,000× *g*) and the resulting supernatants contained the total protein of tissues. Protein samples were mixed with 4X loading buffer supplemented with 2-mercaptoethanol and then boiled at 100 °C for 5 min. Then, 20~40 µg protein was separated by electrophoresis using a 10% sodium dodecyl sulphate-polyacrylamide gel along with a molecular weight marker (100 V, 120 min) and then transferred to a methanol-activated polyvinylidene difluoride (PVDF, Bio-Rad Laboratories, Hercules, CA, USA) membrane by wet transfer (100 V, 90 min). The PVDF membrane was blocked at room temperature for 1 h using 5% (*w*/*v*) bovine serum albumin (BSA) in 0.1% Tris-buffered saline-Tween 20 solution (TBST). Subsequently, the membrane was incubated with appropriate dilutions of primary antibodies targeting NK1R, Nrf2, Gpx4, and GAPDH (Table 1) overnight at 4 °C with gentle agitation. The membrane was washed with TBST and then incubated with HRP-conjugated secondary antibodies (1:5000) at room temperature for 1 h the following day. After washing with TBST, the membrane was detected with ECL Western blotting substrate solution (Thermo Fisher Scientific, Waltham, MA, USA), followed by visualizing the bands on a UVITec Alliance Q9 Advanced machine (Uvitec Cambridge Ltd., Cambridge, UK). Bands were semi-quantitated by the ImageJ software version 1.54d and compared for relative intensities. Results were expressed as fold increases over the control.

### 2.8. Immunohistochemistry Staining

Formalin-fixed lung tissues were processed using a tissue processor (Leica Biosystems, Wetzlar, Germany) and embedded in paraffin wax using an embedding station (Leica Biosystems, Wetzlar, Germany). Prior to staining, 4 µm tissue sections were prepared using a microtome (Leica Biosystems, Wetzlar, Germany), followed by mounting onto adhesive microscope slides (Trajan Scientific and Medical, Ringwood, Australia). Tissue sections were further deparaffinized and rehydrated. These pre-processed tissue sections underwent an antigen retrieval process completed in a pressure cooker (100 °C, 4 min) with Tris-EDTA buffer (pH 9.0), followed by cooling down to room temperature in the buffer (~two hours). These sections were then permeabilizated by PBS with 0.25% Triton (*w*/*v*) for 15 min at 4 °C. After washing with PBST, tissue sections were blocked with 5% BSA in the permeabilization buffer for 1 h at room temperature, followed by incubation with appropriate dilutions of primary antibodies targeting to Nrf2 and Gpx4 overnight in a humidified chamber (4 °C), as described in Table 1. After 3 washes with PBST, lung sections were incubated with 3% H_2_O_2_ for 15 min to inactivate tissue endogenous peroxidases (room temperature). Subsequently, lung sections were incubated with an appropriate dilution of HRP-conjugated secondary antibody (Table 1) for 1 h (room temperature). After washing, these sections were incubated with DAB solution for 3 min (Nrf2) or 5 min (Gpx4) in the dark (room temperature). Slides were dehydrated, permanently mounted, and observed under a light microscope. The expressions of Nrf2 and Gpx4 were quantitated using ImageJ software version 1.54d (IHC profiler plugin).

### 2.9. Measurement of Iron, Malondialdehyde (MDA), and GSH in Tissues

The concentrations of iron (Universal Biologicals Ltd., Cambridge, UK), MDA (BQC Redox Technologies, Asturias, Spain), and GSH (Cayman Chemical, Ann Arbor, MI, USA) were measured in the liver and lung directly according to the instructions of the corresponding assay kits. The concentrations of these mediators in tissues were corrected for the total protein content of the tissues. The results were presented as fold increases over the control.

### 2.10. Histological Analysis

Pre-processed tissue slides were stained with haematoxylin and eosin and the tissue structure was observed under a light microscope (Leica Biosystems, Wetzlar, Germany). The severity of acute liver injury was measured according to the “Hepatic Injury Severity Scoring”, as described previously [34]. The severity of sepsis-associated acute lung injury was evaluated according to a previously reported semiquantitative scoring system [35].

### 2.11. Statistical Analysis

Data are presented as mean ± SD. GraphPad Prism Software version 9.2.0 (GraphPad Software Incorporated, San Diego, CA, USA) was used to perform all the statistical analyses in this study. All data were analysed for normal distribution by the Shapiro–Wilk test. One-way or Two-way Analysis of Variance (ANOVA) with post hoc Tukey’s multiple comparisons test was performed with the normal distribution data to compare multiple groups. Statistical significance was set as *p*-value < 0.05.

## 3. Results

### 3.1. Suppressing SP-NK1R Signalling Improved the Physical Condition of Mice following CLP Surgery-Induced Sepsis

The typical manifestations of sepsis, including piloerection, shivering, reduced vigilance and movement, lethargy, ocular and nasal discharge, lower food and water consumption, and faecal adhesions on the anus, were observed in the *Tac1*^+/+^ mice following CLP-surgery-induced sepsis. None of these abnormalities were observed in the sham-operated mice. Suppressing the SP-NK1R signalling by either the genetic deletion of the *Tac1* gene (*p* < 0.001), the pharmacological blockade of NK1R using L703606 (*p* < 0.001), or the combination of these two methods (*p* < 0.001) significantly mitigated these abnormalities in mice with CLP-surgery-induced sepsis, leading to better a physical condition and lower sepsis severity scores (Table 2). Notably, there was no significant difference in the sepsis severity scores of mice among these three groups (*p* > 0.05).

**Figure 2:**
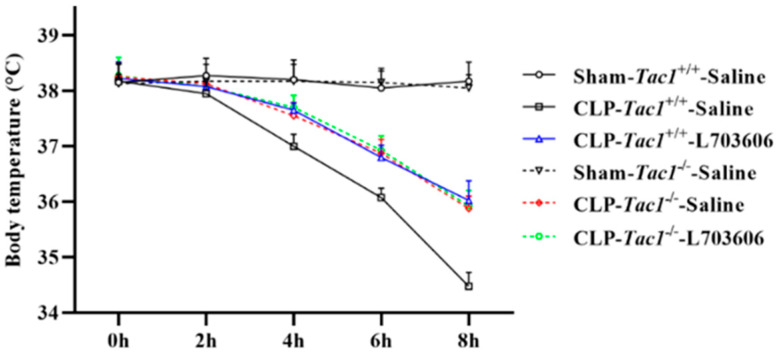
Changes in the body temperature in mice. The body temperature of mice significantly decreased two hours after CLP surgery. Suppressing SP-NK1R signalling attenuated CLP-surgery-induced hypothermia in mice. Data were presented as mean ± SD (n = 4).

As shown in Figure 2, the mean body temperatures decreased significantly four hours after CLP surgery (*p* < 0.001). Suppressing the SP-NK1R axis through either the genetic deletion of the *Tac1* gene (*p* < 0.001), the pharmacological blockade of NK1R using L703606 (*p* < 0.001), or the combination of these two methods (*p* < 0.001) significantly attenuated sepsis-associated hypothermia in mice four hours, six hours, and eight hours after CLP surgery (*p* < 0.01). Notably, there was no significant difference in the body temperature of mice among these three groups (*p* > 0.05). These results collectively indicate that SP-NK1R signalling suppression improved the physical condition of mice with CLP-surgery-induced sepsis.

### 3.2. Concentration of SP in the Liver and Lungs in Mice

As shown in Table 3, compared with the sham operation, CLP surgery led to a significant increase in the concentration of SP in the liver (*p* < 0.001) and lungs (*p* < 0.001) in the *Tac1*^+/+^ mice. Blocking the interaction between SP and NK1R using L703606 attenuated CLP surgery induced an increase in the concentration of SP in the liver (*p* < 0.001) and lungs (*p* < 0.001) in the *Tac1*^+/+^ mice. As expected, SP was not detectable in the liver and lungs in the *Tac1*^−/−^ mice using the SP ELISA kit, irrespective of sham operation and CLP surgery.

### 3.3. Expression of NK1R in the Liver and Lungs in Mice

**Figure 3:**
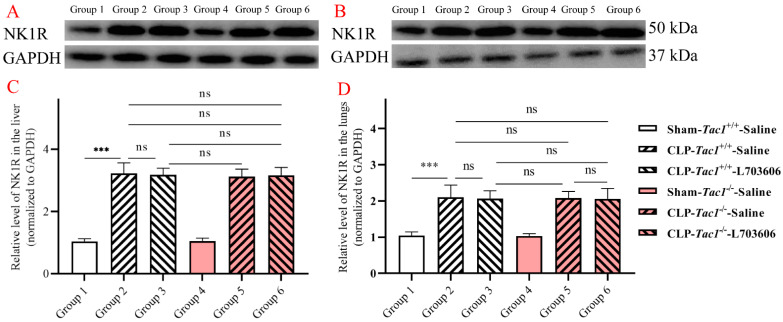
Expressions of NK1R in the liver and lungs in mice. CLP surgery increased the expression of NK1R in the liver (**A**,**C**) and lungs (**B**,**D**) in mice. Disrupting the SP-NK1R axis failed to affect NK1R expression in these tissues in mice with CLP-surgery-induced sepsis. *** *p* < 0.001, ns: Not significant. Data were presented as mean ± SD (n = 6).

As depicted in Figure 3, the expression of NK1R in the liver (*p* < 0.001) and lungs (*p* < 0.001) was significantly elevated in the *Tac1*^+/+^ mice with CLP-surgery-induced sepsis compared with the sham-operated controls. The expression of NK1R in the liver (*p* > 0.05) and lungs (*p* > 0.05) between the *Tac1*^+/+^ mice and the *Tac1*^−/−^ mice in the sham-operated group was not significantly different. Interestingly, disrupting the SP-NK1R axis through either the genetic deletion of the *Tac1* gene, the pharmacological blockade of NK1R using L703606, or the combination of these two approaches had no significant effects on the expressions of NK1R in the liver (*p* > 0.05) and lungs (*p* > 0.05) in mice following CLP-surgery-induced sepsis. This unexpected result suggests that other factors or pathways may be influencing the concentration of NK1R in the liver and lungs in mice with sepsis.

### 3.4. Suppressing SP-NK1R Signalling Attenuated the Increase in the Concentrations of IL-1β, IL-6, and TNF-α in the Liver and Lungs in Mice following CLP-Surgery-Induced Sepsis

**Figure 4:**
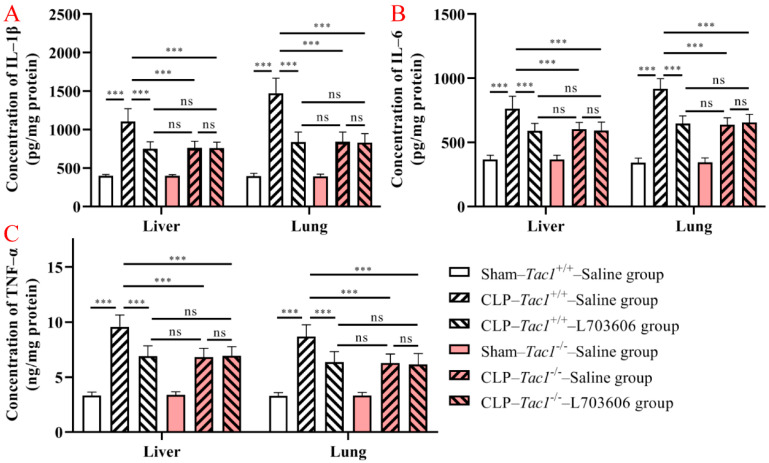
Concentrations of IL-1β, IL-6, and TNF-α in the liver and lungs in mice. CLP surgery increased the concentrations of IL-1β (**A**), IL-6 (**B**), and TNF-α (**C**) in the liver and lungs in mice. Suppressing the SP-NK1R axis attenuated CLP-surgery-induced elevation in the concentrations of IL-1β, IL-6, and TNF-α in these tissues. *** *p* < 0.001, ns: Not significant. Data were presented as mean ± SD (n = 8).

As shown in Figure 4, the concentrations of IL-1β (*p* < 0.001), IL-6 (*p* < 0.001), and TNF-α (*p* < 0.001) were significantly elevated in the liver in the *Tac1*^+/+^ mice following CLP-surgery-induced sepsis compared with the sham-operated controls. In contrast, the increase in the concentrations of IL-1β (*p* < 0.001), IL-6 (*p* < 0.001), and TNF-α (*p* < 0.001) in the liver was attenuated by disrupting the SP-NK1R axis through either the deletion of the *Tac1* gene, the pharmacological blockade of NK1R using L703606, or the combination of these two approaches. Notably, there was no significant difference in the expression of IL-1β (*p* > 0.05), IL-6 (*p* > 0.05), and TNF-α (*p* > 0.05) in the liver in mice among these three groups. As Figure 4 indicates, the changes in the concentrations of IL-1β, IL-6, and TNF-α in the lungs in mice followed a similar pattern observed in the liver. These results show that SP-NK1R signalling suppression attenuated the CLP-surgery-induced increase in the concentrations of IL-1β, IL-6, and TNF-α in the liver and lungs in mice.

### 3.5. Suppressing SP-NK1R Signalling Attenuated the Increase in the Concentrations of MCP-1 and MIP-2 in the Liver and Lungs in Mice following CLP-Surgery-Induced Sepsis

**Figure 5:**
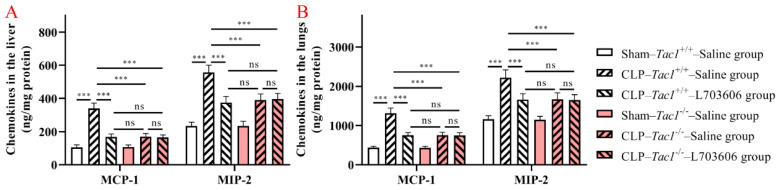
Concentrations of MCP-1 and MIP-2 in the liver and lungs in mice with CLP-surgery-induced sepsis. CLP surgery increased the concentrations of MCP-1 and MIP-2 in the liver (**A**) and lungs (**B**). Suppressing the SP-NK1R axis attenuated CLP-surgery-induced elevation in the concentrations of MCP-1 and MIP-2 in these tissues. *** *p* < 0.001, ns: Not significant. Data were presented as mean ± SD (n = 8).

As shown in Figure 5, the concentrations of MCP-1 (*p* < 0.001) and MIP-2 (*p* < 0.001) were significantly increased in the liver in the *Tac1*^+/+^ mice in comparison with the sham-operated controls. In contrast, the increases in the concentrations of MCP-1 (*p* < 0.001) and MIP-2 (*p* < 0.001) in the liver were attenuated by disrupting the SP-NK1R signalling through either the deletion of the *Tac1* gene, the pharmacological blockade of NK1R using L703606, or the combination of these two approaches. Notably, there was no significant difference in expressions of MCP-1 (*p* > 0.05) and MIP-2 (*p* > 0.05) in the liver in mice among these three groups. As Figure 4 indicates, the changes in the concentration of MCP-1 and MIP-2 in the lungs of the *Tac1*^+/+^ mice with CLP-surgery-induced sepsis followed a similar trend observed in the liver. These results demonstrate that SP-NK1R signalling suppression attenuated the CLP-surgery-induced increase in the concentrations of MCP-1 and MIP-2 in the liver and lungs in mice.

### 3.6. Suppressing SP-NK1R Signalling Attenuated the Increase in the Concentrations of Iron in the Liver and Lungs in Mice following CLP-Surgery-Induced Sepsis

As shown in Table 4, the concentration of iron in the liver was significantly increased in the *Tac1*^+/+^ mice with CLP-surgery-induced sepsis compared with the sham-operated controls (both the *Tac1*^+/+^ mice and the *Tac1*^−/−^ mice, *p* < 0.001). In contrast, suppressing SP-NK1R signalling through either the deletion of the *Tac1* gene (*p* < 0.01), the pharmacological blockade of NK1R using L703606 (*p* < 0.001), or the combination of these two approaches (*p* < 0.001) attenuated the increase in the concentration of iron in the liver in mice with CLP-surgery-induced sepsis. Notably, there was no significant difference in the accumulation of iron in the liver in mice among these three groups (*p* > 0.05). Additionally, as Table 4 indicates, the changes in the accumulation of iron in the lungs in mice with CLP-surgery-induced sepsis followed a similar trend observed in the liver, described above. These results indicate that SP-NK1R signalling suppression attenuated the CLP-surgery-induced increase in the concentrations of iron in the liver and lungs in mice.

### 3.7. Suppressing SP-NK1R Signalling Attenuated the Increase in the Concentration of MDA in the Liver and Lungs in Mice following CLP-Surgery-Induced Sepsis

As shown in Table 5, the concentration of MDA in the liver was significantly increased in the *Tac1*^+/+^ mice with CLP-surgery-induced sepsis compared with the sham-operated controls (*p* < 0.001). In contrast, suppressing the SP-NK1R axis through either the deletion of the *Tac1* gene (*p* < 0.01), the pharmacological blockade of NK1R using L703606 (*p* < 0.001), or the combination of these two approaches (*p* < 0.001) attenuated the increase in the production of MDA in the liver in mice with CLP-surgery-induced sepsis. Notably, there was no significant difference in the concentration of MDA in the liver in mice among these three groups (*p* > 0.05). In addition, as Table 5 indicates, the changes in the production of MDA in the lungs in mice with CLP-surgery-induced sepsis followed a similar pattern to that observed in the liver described above. These results show that SP-NK1R signalling suppression attenuated a CLP-surgery-induced increase in the concentrations of MDA in the liver and lungs in mice.

### 3.8. Suppressing SP-NK1R Signalling Attenuated the Decrease in the Concentration of GSH in the Liver and Lungs in Mice following CLP-Surgery-Induced Sepsis

As shown in Table 6, in contrast to the changes in the concentration of iron and MDA found in the liver and lungs, *Tac1*^+/+^ mice with CLP-surgery-induced sepsis showed lower concentrations of GSH in the liver (*p* < 0.001) and lungs (*p* < 0.001) than the sham-operated controls. Suppressing the SP-NK1R axis through either the genetic deletion of the *Tac1* gene (*p* < 0.001), the pharmacological blockade of NK1R using L703606 (*p* < 0.001), or the combination of these two approaches (*p* < 0.001) attenuated the decrease in the concentration of GSH in the liver and lungs in mice with CLP-surgery-induced sepsis. Notably, there was no significant difference in the concentration of GSH in the liver (*p* > 0.05) and lungs (*p* > 0.05) in mice among these three groups. These results demonstrate that SP-NK1R signalling suppression attenuated the CLP-surgery-induced decrease in the concentration of GSH in the liver and lungs in mice.

### 3.9. Suppressing SP-NK1R Signalling Attenuated the Decrease in the Expressions of Nrf2 and Gpx4 in the Liver in Mice following CLP-Surgery-Induced Sepsis

**Figure 6:**
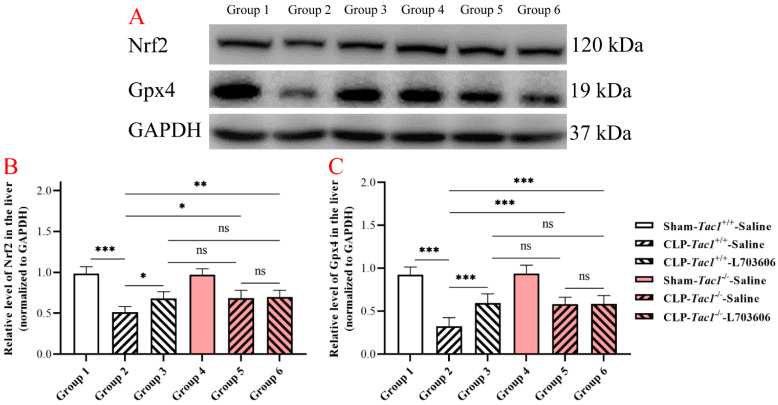
The expressions of Nrf2 and Gpx4 in the liver in mice with CLP-surgery-induced sepsis. Representative Western blotting images of Nrf2, Gpx4, and GAPDH in the liver are shown in panel (**A**). CLP surgery significantly decreased the expressions of the Nrf2 panel (**B**) and Gpx4 panel (**C**) in the liver in the *Tac1*^+/+^ mice. These alterations were attenuated by suppressing the SP-NK1R axis. * *p* < 0.05, ** *p* < 0.01, *** *p* < 0.001; ns: not significant. Data were presented as mean ± SD (n = 6).

As shown in Figure 6, the concentration of Nrf2 in the liver was significantly reduced in the *Tac1*^+/+^ mice with CLP-surgery-induced sepsis compared with the sham-operated controls (*p* < 0.001). Suppressing the SP-NK1R axis through either the deletion of the *Tac1* gene (*p* < 0.05), the pharmacological blockade of NK1R using L703606 (*p* < 0.05), or the combination of these two approaches (*p* < 0.01) attenuated the decrease in the expression of Nrf2 in the liver in mice with CLP-surgery-induced sepsis. Notably, there was no significant difference in the expression of Nrf2 in the liver in mice among these three groups (*p* > 0.05). Similarly, the expression of Gpx4 in the liver in the *Tac1*^+/+^ mice with CLP-surgery-induced sepsis was lower than in the sham-operated controls (*p* < 0.001). In contrast, suppressing the SP-NK1R axis through either the genetic deletion of the *Tac1* gene (*p* < 0.05), the pharmacological blockade of NK1R using L703606 (*p* < 0.05), or the combination of these two approaches (*p* < 0.01) attenuated the decrease in the expression of Gpx4 in the liver in the *Tac1*^+/+^ mice following CLP-surgery-induced sepsis. There was no significant difference in the expression of Nrf2 (*p* > 0.05) and Gpx4 (*p* > 0.05) in the liver in mice among these three groups. These results indicate that SP-NK1R signalling suppression attenuated the CLP-surgery-induced decrease in the concentrations of Nrf2 and Gpx4 in the liver in mice.

### 3.10. Suppressing SP-NK1R Signalling Attenuated the Decreases in the Expressions of Nrf2 and Gpx4 in the Lungs in Mice following CLP-Surgery-Induced Sepsis

**Figure 7:**
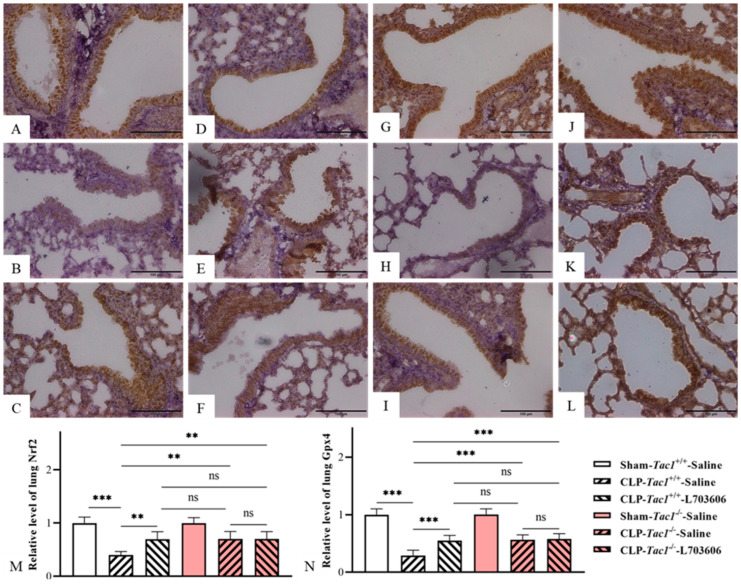
The expression of Nrf2 and Gpx4 in the lungs of mice with CLP-surgery-induced sepsis. Representative IHC images of lung Nrf2 and Gpx4 from each group are presented in panel (**A**–**F**) and panel (**G**–**L**), respectively. The expressions of lung Nrf2 and Gpx4 (Brown staining) are plotted as the fold increase over the sham-operated control and shown in panels M and N, respectively. CLP surgery significantly decreased the expressions of Nrf2 and Gpx4 in the lungs in the *Tac1*^+/+^ mice. These alterations were attenuated by suppressing the SP-NK1R axis. ** *p* < 0.01, *** *p* < 0.001; ns: not significant. Data were presented as mean ± SD (n = 6).

In accordance with the change in the expressions of Nrf2 and Gpx4 in the liver, the *Tac1*^+/+^ mice with CLP-surgery-induced sepsis also showed significantly lower expressions of Nrf2 (*p* < 0.001) and Gpx4 (*p* < 0.001) in the lungs in comparison with the sham-operated controls, as depicted in Figure 7. In contrast, disrupting the SP-NK1R axis through either the genetic deletion of the *Tac1* gene, the pharmacological blockade of NK1R using L703606, or the combination of these two approaches attenuated the decreases in the expression of Nrf2 (*p* < 0.01) and Gpx4 (*p* < 0.001) in the lungs in mice with CLP-surgery-induced sepsis. Notably, there was no significant difference in the expressions of Nrf2 (*p* > 0.05) and Gpx4 (*p* > 0.05) in the lungs in mice among these three groups. These results indicate that SP-NK1R signalling suppression attenuated the CLP-surgery-induced decrease in the concentrations of Nrf2 and Gpx4 in the lungs of mice.

### 3.11. Suppressing SP-NK1R Signalling Mitigated the Severity of Liver Inflammatory Injury in Mice following CLP-Surgery-Induced Sepsis

**Figure 8:**
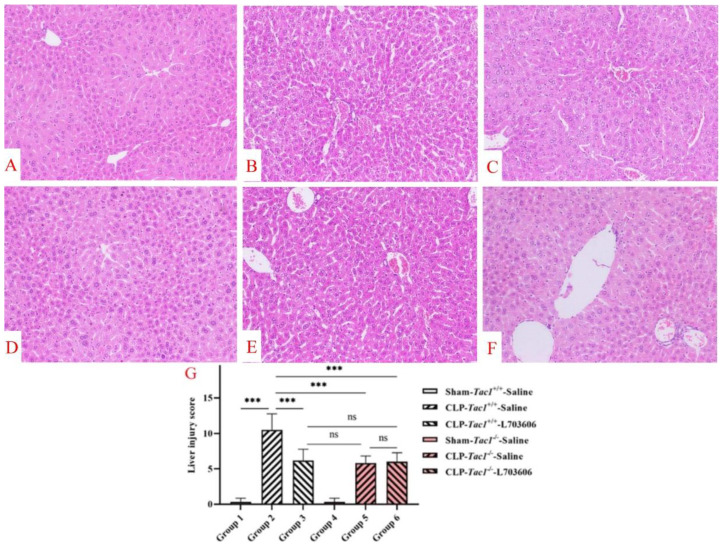
Liver structural damage in mice with CLP-surgery-induced sepsis. Representative images of liver structures are shown in panels A–F. While the liver structures of the *Tac1*^+/+^ mice (**A**) and the *Tac1*^−/−^ mice (**D**) in the sham-operated group were normal, those of the *Tac1*^+/+^ mice with CLP-surgery-induced sepsis were damaged (**B**). These abnormalities in mice were attenuated by disrupting the SP-NK1R axis (**C**,**E**,**F**). Liver injury scores are shown in panel (**G**). *** *p* < 0.001; ns: not significant. Data were presented as mean ± SD (n = 8).

As depicted in Figure 8, the liver structures of mice in the sham-operated groups (both the *Tac1*^+/+^ mice and the *Tac1*^−/−^ mice) were normal and intact. However, CLP surgery led to many abnormalities in the liver structures in the *Tac1*^+/+^ mice, including oedematous hepatocytes, nuclear pyknosis, erythrocyte deposition, and neutrophilic infiltration. In contrast, disrupting the SP-NK1R axis through either the genetic deletion of the *Tac1* gene, the pharmacological blockade of NK1R using L703606, or the combination of these two approaches protected mice against CLP-surgery-induced liver architectural damage. The semiquantitative analysis of these images showed that increased SP-NK1R signalling was positively associated with the severity of liver structural damage (as evidenced by the lung injury scores) in mice following CLP-surgery-induced sepsis. Notably, there was no significant difference in the liver injury scores of mice with CLP-surgery-induced sepsis who underwent any of the following manipulations: the genetic deletion of the *Tac1* gene, the pharmacological blockade of NK1R, or the combination of these two approaches (*p* > 0.05). These results indicate that SP-NK1R signalling suppression attenuated CLP-surgery-induced acute inflammatory injury in the liver in mice.

### 3.12. Suppressing SP-NK1R Signalling Mitigated the Severity of Lung Inflammatory Injury in Mice following CLP-Surgery-Induced Sepsis

**Figure 9:**
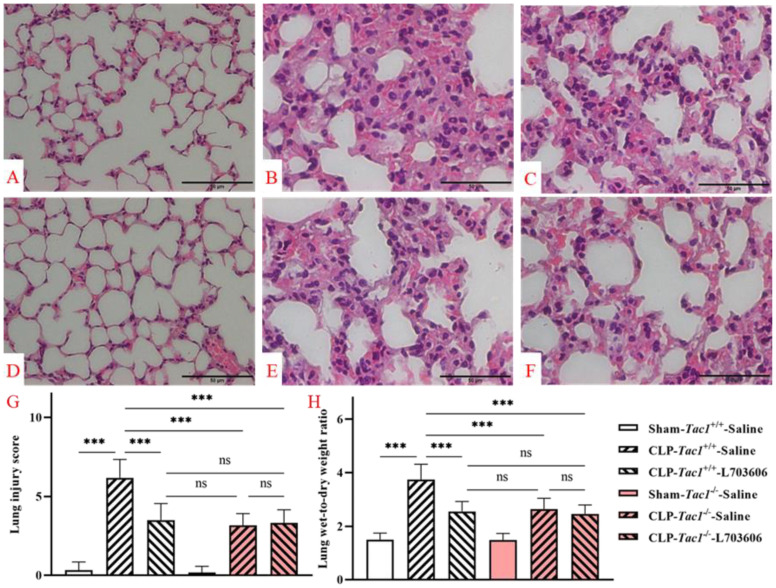
Lung structural damage in mice following CLP-surgery-induced sepsis. Representative images of lung structures are shown in panels (**A**–**F**). While the lung structures of the *Tac1*^+/+^ mice (**A**) and the *Tac1*^−/−^ mice (**D**) in the sham-operated group were normal, those of the *Tac1*^+/+^ mice with CLP surgery-induced sepsis were damaged (**B**). These abnormalities in mice were attenuated by disrupting the SP-NK1R axis (**C**,**E**,**F**). Lung injury score and W/D ratio are shown in panels (**G**,**H**), respectively. *** *p* < 0.001; ns: not significant. Data were presented as mean ± SD (n = 8).

As depicted in Figure 9, the lung structures of mice in the sham-operated groups (both the *Tac1*^+/+^ mice and the *Tac1*^−/−^ mice) were normal and intact. However, CLP surgery led to many abnormalities in the lung structures in the *Tac1*^+/+^ mice, such as inflammatory cell infiltration, alveolar septal thickening, interstitial oedema, and haemorrhage. In contrast, disrupting the SP-NK1R axis through either the genetic deletion of the *Tac1* gene, the pharmacological blockade of NK1R using L703606, or the combination of these two approaches protected mice against CLP-surgery-induced lung architectural damage in mice. The semiquantitative analysis of these images showed that the increase in SP-NK1R signalling was positively associated with the severity of lung structural damage (as evidenced by the lung injury scores), particularly pulmonary oedema (as evidenced by the lung W/D ratio), in mice following CLP-surgery-induced sepsis. Notably, there was no significant difference in the lung injury scores (*p* > 0.05) and the lung W/D ratios (*p* > 0.05) of mice following CLP-surgery-induced sepsis who underwent any of the following manipulations: the genetic deletion of the *Tac1* gene, the pharmacological blockade of NK1R, or the combination of these two approaches. These results indicate that SP-NK1R signalling suppression attenuated CLP-surgery-induced acute inflammatory injury in the lungs of mice.

## 4. Discussion

The pathogenesis of dysregulated acute inflammatory reaction in sepsis involves many mechanisms [3,36]. As an immune-derived cell death, ferroptosis is also closely associated with the dysregulation of host acute inflammatory responses under various pathological conditions [12,24,25]. Moreover, dysregulated ferroptosis has been linked to the physiopathological mechanism of dysregulated acute inflammatory response in sepsis and related organ injury [14,15,16]. This study demonstrated that CLP surgery significantly activated SP-NK1R signalling in the liver and lungs, leading to increased ferroptosis in these tissues in mice. In contrast, suppressing the increased SP-NK1R signalling attenuated these alterations in mice. This protection effect was accompanied by an attenuated acute inflammatory response (pro-inflammatory cytokines and chemokines) and injury (architectural destructions) in the liver and lungs and clinical signs (hypothermia, piloerection, and shivering) in mice and improved the physical conditions of mice (sepsis severity scores). This study also demonstrated that the CLP surgery inactivated Nrf2-Gpx4/GSH signalling, thereby promoting ferroptosis in the liver and lungs in mice, which was associated with the detrimental impact of the SP-NK1R axis in sepsis.

Ferroptosis is a recently recognized programmed cell death resulting from the increased accumulation of intracellular iron-mediated dysregulation of lipid peroxidation [10]. Many studies have illustrated that aberrant ferroptosis contributes to organ impairment caused by sepsis, whereas inhibiting ferroptosis attenuates sepsis-related organ injury [14,15,16]. Intracellular iron overload and increased lipid peroxidation are two well-recognized markers of ferroptosis [10,37]. In line with previous investigations, this study also observed a significant elevation in the concentration of iron in the liver and lungs in mice following CLP-surgery-induced sepsis [38,39]. MDA is a crucial final product of the process of lipid peroxidation; thus, MDA concentration is a frequently used index of lipid peroxidation [40]. Consistent with previous investigations, we found that the concentration of MDA in the liver and lungs in mice with CLP-surgery-induced sepsis was also increased [38,39]. These alterations show that the dysregulation of intracellular iron accumulation and lipid peroxidation in the liver and lungs in mice arises following CLP-surgery-induced sepsis, indicating that ferroptosis was increased in these tissues in mice with sepsis. Given the roles of the SP-NK1R axis in promoting acute inflammatory response [5,41,42] and COX-2 expression [27,28,29] in inflammation-related pathological circumstances, it is worth probing into the impact of this axis on the profile of ferroptosis in sepsis. Notably, this study showed that these increases in the concentration of iron and MDA in the liver and lungs in mice following CLP-surgery-induced sepsis were reduced by either the deletion of the *Tac1* gene, the pharmacological blockage of NK1R, or the combination of these two methods. These results collectively suggest that increased SP-NK1R signalling contributes to ferroptosis in the liver and lungs in mice following CLP-surgery-induced sepsis. Notably, SP-NK1R signalling has a similar impact on the acute inflammatory response and injury in the liver and lungs in mice following CLP-surgery-induced sepsis, indicating that elevated ferroptosis is associated with acute liver and lung injury in sepsis.

The discovery of the role of the SP-NK1R axis in ferroptosis in the liver and lungs in mice with CLP-surgery-induced sepsis and the potential association between ferroptosis and acute liver and lung injury in sepsis prompted us to investigate the underlying mechanisms by which the SP-NK1R axis participates in CLP-surgery-induced ferroptosis. Ferroptosis is a step-wise cell death process involving the dysregulated activation of various signalling cascades [43]. Gpx4 is crucial to the maintenance of lipid homeostasis, suggesting that Gpx4 is involved in ferroptosis [18,43]. Gpx4 is a crucial enzyme in converting toxic lipid hydroperoxide to nontoxic lipid alcohol [18,43]. Thus, Gpx4 has been widely recognized as a cornerstone of lipid antiperoxidative defence and a gatekeeper of ferroptosis [18,43]. This study showed a significant decrease in the expression of Gpx4 in the liver and lungs in mice following CLP-induced sepsis, which agrees with previous research [38,39]. The antioxidant GSH is an essential reducing substrate for Gpx4, and it is also crucial to maintaining the activity of Gpx4. Thus, GSH is also treated as a critical regulator of ferroptosis [18,43]. In line with previous studies, we found that the concentration of GSH in the liver and lungs was significantly downregulated in mice with CLP-surgery-induced sepsis [38,39]. Notably, this study also demonstrated that these decreases in the expression of Gpx4 and GSH in the liver and lungs in mice with CLP-surgery-induced sepsis were reversed by suppressing SP-NK1R signalling. Compelling evidence has pointed to the involvement of transcription factors in ferroptosis [20,43]. It was reported that Nrf2 regulates the expression of several genes involved in iron metabolism; thus, Nrf2 plays a crucial role in maintaining iron homeostasis. Moreover, multiple genes involved in detoxification or antioxidant responses, including Gpx4, and genes that regulate GSH metabolism, are the target genes of Nrf2; therefore, Nrf2 also plays a central role in suppressing lipid peroxidation. The role of Nrf2 in maintaining the homeostasis of iron and lipid makes it a well-recognized inhibitor of ferroptosis [19,20]. In line with previous investigations, the expression of Nrf2 in the liver and lungs was significantly decreased in mice with CLP-surgery-induced sepsis [38,39]. In contrast, the disruption of the SP-NK1R axis reversed the decreases in the expression of Nrf2 in these tissues in mice following CLP-surgery-induced sepsis. These results collectively indicate that increased SP-NK1R signalling inactivates the Nrf2-Gpx4/GSH cascade, thereby promoting ferroptosis in CLP-surgery-induced sepsis.

We also found that NK1R antagonist treatment did not cause any further effect on the profile of ferroptosis as well as acute inflammatory response and injury in the liver and lungs in *Tac1*^−/−^ mice with CLP-surgery-induced sepsis. Moreover, if the NK1R antagonist was utilized to block the actions of NK1R, the deletion of the *Tac1* gene did not cause any further effect on the profile of ferroptosis as well as acute inflammatory response and injury in the liver and lungs in mice with CLP-surgery-induced sepsis. These results suggest that SP contributes to ferroptosis and acute inflammatory responses and injury in the liver and lungs in sepsis via priming NK1R.

This study has some limitations. Firstly, this study was limited to only one time point (8 h after the CLP surgery) and represented alterations emerging at that point only. Another limitation of this study is that the impacts of the SP-NK1R axis on sepsis-associated acute inflammatory response and ferroptosis were only investigated in mice. It would be helpful to investigate the role of this axis in organ impairment and ferroptosis in patients with sepsis, as many differences exist in the pathophysiology of sepsis between mice and humans.

## 5. Conclusions

**Figure 10:**
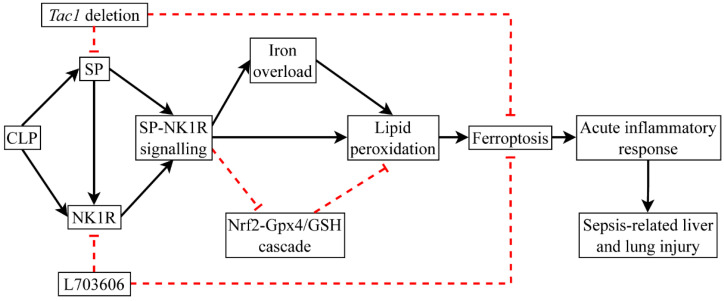
CLP surgery promotes ferroptosis by inactivating the Gpx4/GSH cascade. The detrimental impact of SP on mice with CLP-surgery-induced sepsis is predominantly mediated by NK1R and is associated with increased ferroptosis.

This study demonstrates that CLP surgery inactivated the Nrf2-Gpx4/GSH cascade, leading to elevated ferroptosis in the liver and lungs in mice. This alteration was associated with increased SP-NK1R-signalling-mediated acute inflammatory response and injury in the liver and lungs in mice following CLP-surgery-induced sepsis (Figure 10). This study also demonstrates that NK1R is the primary receptor responsible for the detrimental impact of SP on CLP-surgery-induced ferroptosis and acute inflammatory response and injury in the liver and lungs in mice. Given that SP has emerged as an index of host inflammatory response, sources of infection, and mortality in patients with sepsis, our study could facilitate the development of novel adjuvant treatment for sepsis in clinic, targeting the SP-NK1R cascade.

## Figures and Tables

**Table 1:** Information of antibodies used in this study.

Antibody	Dilution	Source Catalogue No.
NK1R	1:1000	Thermo Fisher Scientific, Waltham, MA, USA
Nrf2	1:1000	Thermo Fisher Scientific, Waltham, MA, USA
Gpx4	1:1000	Thermo Fisher Scientific, Waltham, MA, USA
GAPDH	1:1000	Santa Cruz Biotechnology, Dallas, TX, USA
HRP-conjugated secondary antibody	1:5000	Santa Cruz Biotechnology, Dallas, TX, USA

**Table 2:** Sepsis severity scores of mice.

Group	Sepsis Severity Score
Sham-*Tac1*^+/+^-Saline group	0.63 ± 0.74
CLP-*Tac1*^+/+^-Saline group	9.13 ± 2.17 *
CLP-*Tac1*^+/+^-L703606 group	4.63 ± 1.41 ^#^
Sham-*Tac1*^−/−^-Saline group	0.75 ± 0.71
CLP-*Tac1*^−/−^-Saline group	4.50 ± 1.41 ^#^
CLP-*Tac1*^−/−^-L703606 group	4.88 ± 1.64 ^#^

* *p* < 0.001 versus Sham-*Tac1*^+/+^-Saline group; ^#^ *p* < 0.001 versus CLP-*Tac1*^+/+^-Saline group. Data were presented as mean ± SD (n = 8).

**Table 3:** Concentrations of SP in the liver and lungs in mice.

Group	Tissue SP Concentration (ng/g Protein)	
	Liver	Lung
Sham-*Tac1*^+/+^-Saline group	26.95 ± 2.95	25.13 ± 2.09
CLP-*Tac1*^+/+^-Saline group	390.82 ± 32.40 *	151.46 ± 6.34 *
CLP-*Tac1*^+/+^-L703606 group	237.36 ± 19.83 ^#^	104.17 ± 8.12 ^#^
Sham-*Tac1*^−/−^-Saline group	<the LLOD	<the LLOD
CLP-*Tac1*^−/−^-Saline group	<the LLOD	<the LLOD
CLP-*Tac1*^−/−^-L703606 group	<the LLOD	<the LLOD

* *p* < 0.001 versus Sham-*Tac1*^+/+^-Saline group; ^#^ *p* < 0.001 versus CLP-*Tac1*^+/+^-Saline group. LLOD: Lower limit of detection. Data were presented as mean ± SD (n = 8).

**Table 4:** Concentrations of iron in the liver and lungs in mice.

Group	Tissue Iron Concentration (μg/g Protein)	
	Liver	Lung
Sham-*Tac1*^+/+^-Saline group	2.75 ± 0.19	1.03 ± 0.06
CLP-*Tac1*^+/+^-Saline group	4.33 ± 0.70 *	2.14 ± 0.24 *
CLP-*Tac1*^+/+^-L703606 group	3.51 ± 0.32 ^#^	1.53 ± 0.15 ^&^
Sham-*Tac1*^−/−^-Saline group	2.77 ± 0.17	1.02 ± 0.07
CLP-*Tac1*^−/−^-Saline group	3.48 ± 0.31 ^&^	1.58 ± 0.15 ^&^
CLP-*Tac1*^−/−^-L703606 group	3.40 ± 0.33 ^&^	1.57 ± 0.14 ^&^

* *p* < 0.001 versus Sham-*Tac1*^+/+^-Saline group, ^#^ *p* < 0.01 versus CLP-*Tac1*^+/+^-Saline group, ^&^ *p* < 0.001 versus CLP-*Tac1*^+/+^-Saline group. Data were presented as mean ± SD (n = 8).

**Table 5:** Concentrations of MDA in the liver and lungs in mice.

Group	Tissue MDA Concentration (μg/g Protein)	
	Liver	Lung
Sham-*Tac1*^+/+^-Saline group	2.467 ± 0.15	5.070 ± 0.29
CLP-*Tac1*^+/+^-Saline group	6.297 ± 1.01 *	15.994 ± 1.82 *
CLP-*Tac1*^+/+^-L703606 group	4.947 ± 0.68 ^&^	10.714 ± 1.14 ^&^
Sham-*Tac1*^−/−^-Saline group	2.446 ± 0.17	5.117 ± 0.32
CLP-*Tac1*^−/−^-Saline group	5.065 ± 0.53 ^#^	10.888 ± 0.86 ^&^
CLP-*Tac1*^−/−^-L703606 group	4.985 ± 0.52 ^&^	11.0492 ± 1.08 ^&^

* *p* < 0.001 versus Sham-*Tac1*^+/+^-Saline group, ^#^ *p* < 0.01 versus CLP-*Tac1*^+/+^-Saline group, ^&^ *p* < 0.001 versus CLP-*Tac1*^+/+^-Saline group. Data were presented as mean ± SD (n=8).

**Table 6:** Concentrations of GSH in the liver and lungs in mice.

Group	Tissue GSH Concentration (μg/mg Protein)	
	Liver	Lung
Sham-*Tac1*^+/+^-Saline group	29.60 ± 1.52	2.47 ± 0.10
CLP-*Tac1*^+/+^-Saline group	17.63 ± 2.84 *	1.08 ± 0.20 *
CLP-*Tac1*^+/+^-L703606 group	24.20 ± 2.08 ^&^	2.10 ± 0.17 ^&^
Sham-*Tac1*^−/−^-Saline group	29.72 ± 1.53	2.47 ± 0.11
CLP-*Tac1*^−/−^-Saline group	24.33 ± 1.70 ^&^	2.06 ± 0.14 ^&^
CLP-*Tac1*^−/−^-L703606 group	24.67 ± 1.63 ^&^	2.03 ± 0.14 ^&^

* *p* < 0.001 versus Sham-*Tac1*^+/+^-Saline group, ^&^ *p* < 0.001 versus CLP-*Tac1*^+/^-Saline group. Data were presented as mean ± SD (n = 8).

## Data Availability

All data generated or analysed during this study are included in this published article.
